# Attempting genetic inference from directional asymmetry during convergent hindlimb reduction in squamates

**DOI:** 10.1002/ece3.9088

**Published:** 2022-07-14

**Authors:** Samantha Swank, Ethan Elazegui, Sophia Janidlo, Thomas J. Sanger, Michael A. Bell, Yoel E. Stuart

**Affiliations:** ^1^ Department of Biology Loyola University Chicago Chicago Illinois USA; ^2^ Committee on Development, Regeneration, and Stem Cell Biology University of Chicago Chicago Illinois USA; ^3^ UC Museum of Paleontology University of California Berkeley California USA

**Keywords:** development, hindlimb loss, micro‐computed tomography, museum specimens, parallel evolution, *Pitx1*

## Abstract

Loss and reduction in paired appendages are common in vertebrate evolution. How often does such convergent evolution depend on similar developmental and genetic pathways? For example, many populations of the threespine stickleback and ninespine stickleback (Gasterosteidae) have independently evolved pelvic reduction, usually based on independent mutations that caused reduced *Pitx1* expression. Reduced *Pitx1* expression has also been implicated in pelvic reduction in manatees. Thus, hindlimb reduction stemming from reduced *Pitx1* expression has arisen independently in groups that diverged tens to hundreds of millions of years ago, suggesting a potential for repeated use of *Pitx1* across vertebrates. Notably, hindlimb reduction based on the reduction in *Pitx1* expression produces left‐larger directional asymmetry in the vestiges. We used this phenotypic signature as a genetic proxy, testing for hindlimb directional asymmetry in six genera of squamate reptiles that independently evolved hindlimb reduction and for which genetic and developmental tools are not yet developed: *Agamodon anguliceps*, *Bachia intermedia*, *Chalcides sepsoides*, *Indotyphlops braminus*, *Ophisaurus attenuatuas and O. ventralis*, and *Teius teyou*. Significant asymmetry occurred in one taxon, *Chalcides sepsoides*, whose left‐side pelvis and femur vestiges were 18% and 64% larger than right‐side vestiges, respectively, suggesting modification in *Pitx1* expression in that species*.* However, there was either right‐larger asymmetry or no directional asymmetry in the other five taxa, suggesting multiple developmental genetic pathways to hindlimb reduction in squamates and the vertebrates more generally.

## Graphical abstract

We quantified directional asymmetry in the vestigial hindlimbs of six squamate lineages to test for parallel loss of gene expression of Pitx1 during convergent limb reduction. We found left‐larger asymmetry in one of six taxa, suggesting that Pitx1 may be involved in that lineage. However, it is likely that non‐parallel genetic mechanisms underlie pelvic reduction in the squamates.

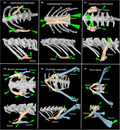

## 1 INTRODUCTION

To what extent is repeated phenotypic evolution caused by a change to the same developmental genetic pathways (Losos, 2011; Bolnick, 2018)? For example, appendage reduction has been a major theme in vertebrate evolution. How often are the same developmental genetic pathways involved in phylogenetically independent instances of appendage reduction?

Independent evolution of pelvic reduction has been reported in many populations of Threespine Stickleback fish (*Gasterosteus aculeatus*) (Klepaker et al., 2013). The repeated reduction has often depended on similar but independent de novo deletion mutations in regulatory regions of the transcription factor *Pitx1*, severely reducing or eliminating expression in the developing pelvic region and resulting in pelvic reduction (Bell et al., 2007; Chan et al., 2010; Cole et al., 2003; Coyle et al., 2007; Shapiro et al., 2004, 2006; Thompson et al., 2018; Xie et al., 2019). Thus, among *G. aculeatus* populations, silencing *Pitx1* provides a recurrent molecular route to similar phenotypic outcomes. Repeated pelvic reduction in ninespine stickleback, *Pungitius pungitius*, and its sister taxon, *Culaea inconstans* (Brook Stickleback), may also depend on independent reduction in *Pitx1* expression (Nelson, 1971; Nelson & Atton, 1971; Shapiro et al., 2004, 2006; Xie et al., 2019).

Full development of vertebrate hind appendages requires normal expression of *Pitx1* (Lanctôt et al., 1999; Marcil et al., 2003; Szeto et al., 1999). Eliminating the expression of a functional *Pitx1* molecule in the developing hind appendage results in limb reduction or loss (Chan et al., 2010; Lanctot et al., 1999; Szeto et al., 1999; Thompson et al., 2018). Notably, *Pitx1*‐mediated pelvic reduction is associated with a tendency for the left pelvic vestige to be larger than the right or even to be present in the absence of the right vestige. Asymmetry biased in one direction (e.g., handedness in humans) is called directional asymmetry (Van Valen, 1962). Left‐larger directional asymmetry is present in most threespine and ninespine stickleback populations with reduced pelvises (Bell et al., 1985, 1993, 2007; Shapiro et al., 2004, 2006). In experimental populations of lab mice and chickens, induced mutations of *Pitx1* led to left‐larger asymmetry (Lanctôt et al., 1999; Marcil et al., 2003; Szeto et al., 1999). Left‐larger asymmetry was also observed in humans with reduced *Pitx1* function (Gurnett et al., 2008; Kragesteen et al., 2018). Such asymmetry arises because the absence of a functional *Pitx1* product during hindlimb development unmasks the phenotypic effects of expression of *Pitx2*, a paralog that influences pelvic development and is expressed preferentially on the left side during development (Marcil et al., 2003; Palmer, 2004). Asymmetrical gene expression is rare in vertebrates (Palmer, 2004), making left‐larger pelvic asymmetry a strong phenotypic indicator of reduced expression of *Pitx1* that can be measured inexpensively in large samples of non‐model species without genetic tools.

For example, based on such asymmetry, it was inferred that pelvic reduction in an extinct, threespine stickleback, *G. doryssus*, from the Miocene, 10 million years ago, probably also depended on eliminating *Pitx1* expression (Stuart et al., 2020). If true, manipulation of *Pitx1* expression for pelvic reduction spans about 26 million years of gasterosteid evolution (Varadharajan et al., 2019); a shared pathway at the family level. Phenotypic evidence (i.e., left‐larger pelvic vestiges) also suggests that reduced *Pitx1* expression causes pelvic reduction in the manatee, *Trichechus manatus latirostris* (Shapiro et al., 2006), which has a most recent common ancestor with sticklebacks about 435 million years ago (timetree.org, 2022).

*Pitx1* may therefore be a candidate gene for repeated use during the evolution of hind appendage reduction in vertebrates because its expression is apparently conserved throughout the clade, and it has a large effect on hind appendage development (Gurnett et al., 2008; Kragesteen et al., 2018; Lanctôt et al., 1999; Marcil et al., 2003; Palmer, 2004; Shapiro et al., 2004, 2006; Szeto et al., 1999; Thompson et al., 2018). Furthermore, there are multiple enhancers controlling *Pitx1* expression (including one with elevated frequencies of deletion mutations [Xie et al., 2019]), allowing for pelvis‐specific modulation in expression without affecting expression in other tissues (Chan et al., 2010; Kragesteen et al., 2018; Thompson et al., 2018; Xie et al., 2019). As such, *Pitx1* expression in the hind appendage might evolve quickly by natural selection with minimal constraint from pleiotropic effects.

Nevertheless, a recent review of molecular and developmental evidence from multiple clades of non‐model vertebrates with hind appendage reduction found that *Pitx1* use appears to be limited to sticklebacks and manatees (Swank et al., 2021). Moreover, a recent comparative genomics study of three limb‐reduced reptile lineages found that the molecular basis of limb loss was not convergent and in no lineage was *Pitx1* a major driver (Roscito et al., 2022). In this study, we corroborate the findings of Swank et al. (2021) and Roscito et al. (2022) with new data collected from six lizard clades that independently evolved hindlimb reduction. We tested for *Pitx1*’s signature of left‐larger asymmetry in vestigial hindlimbs and found left‐larger asymmetry in only one species. This suggests that for lizards too, repeated evolution of limb reduction has diverse genetic causes and that *Pitx1* may only be one of many routes to reduction.

## 2 MATERIALS AND METHODS

### 2.1 Power analysis

A hypothesis of *Pitx1* causation in pelvic girdle and hindlimb reduction predicts that a greater number of individuals in a sample will have larger hindlimb vestiges on the left side of the body than on the right. Previous work showed that the ability to detect a bias in directional asymmetry in 33 samples of threespine stickleback with pelvic reduction apparently depended on the frequency of asymmetry and sample size (Bell et al., 2007). To determine the sample size necessary to detect significant deviation from 50:50 left–right asymmetry, we conducted a power analysis in the statistical software R (RCoreTeam, 2021), using the *pwr.chisq.test* function in the package *pwr* (Champely, 2020). For a significance level of 0.05 and 1 degree of freedom, we input effect sizes between 0.0 (no deviation from equal left‐versus‐right bias) and 0.5, increasing in units of 0.05. For a moderate‐effect size of 0.25, sample sizes greater than 60 are needed to achieve a power greater than 0.50, so we sought samples of at least 80 specimens per taxon.

### 2.2 Taxon choice

Squamate reptiles (e.g., lizards and snakes) are appealing subjects to study the presence and repeatability of directional asymmetry in vestigial hindlimbs because hindlimb reduction has evolved at least 26 times within the group (Brandley et al., 2008; Wiens et al., 2006). Taxon choice within squamates was constrained by the availability of museum specimens. We picked six genera for which there are large museum collections that included at least 80 adult individuals per taxon (Table 1). We used adults to assure complete ossification of limb bones and to standardize our comparisons in case the allometric relationship between asymmetry and size changes during development. Each of these six taxa was nested within clades in which full limb development was plesiomorphic and, therefore, represented clear cases of independent hindlimb reduction (Brandley et al., 2008). We chose one species per genus, except for *Ophisaurus*, for which we pooled observations from two species, *O. attenuates* and *O. ventralis* (hereafter *Ophisaurus* spp.) (Wiens & Slingluff, 2001). *Agamodon anguliceps*, an amphisbaenian, *Ophisaurus* spp., anguids, and *Indotyphlops braminus*, a snake, retain vestigial pelvises only. *Bachia intermedia* is a Gymophthalmid with a reduction in relative hindlimb size and digit number (Kohlsdorf & Wagner, 2006; Roscito et al., 2014). *Chalcides sepsoides* is a skink with reduction in relative hindlimb size (A. Resetar, *pers. Comm*). *Teius teyou* is a teiid with a severely reduced, vestigial digit five in the hindlimb (Brandley et al., 2008).

**TABLE 1:** Species, sample sizes, *μ*‐CT parameters, and bones present

Species	Sample size	Voltage (kV)	Current (mA)	Pelvis present	Femur present
*Agamodon anguliceps*	126	65–70	100–114	Yes	No
*Bachia intermedia*	84	70	100	Yes	Yes
*Indotyphlops braminus*	95	70	100	Yes	No
*Ophisaurus* spp.^a^	94	70	100	Yes	No
*Chalcides sepsoides*	90	55	140	Yes	Yes
*Teius teyou*	73	70	100	Yes	Yes

^a^ Pooled specimens from *O. attenuates* (*N* = 74) and *O. ventralis* (*N* = 20). See Supplementary Information for the MuseumSquamateLoansSpreadsheet.xlsx, which lists the museums of origin as well as specimen IDs.

### 2.3 Micro‐computed tomography (*μ*‐CT) scanning

Each specimen was wrapped in plastic and scanned for 4 min using a Perkins‐Elmer Quantum GX2 micro‐CT Imaging System. To reduce image artifacts like beam hardening, we used either an Al 1.0 mm or an Al 0.5 mm + Cu 0.06 mm filter, as needed. Because individual size varied within and among taxa, we adjusted voltage, current, and voxel size for specimen size (Table 1). We used the smallest voxel size that still contained the pelvis and femurs (if present).

### 2.4 Image processing

We used the open‐source program InVesalius v.3.1 to reconstruct 3D images from raw DICOM files. We applied a reconstruction threshold to reduce image noise. Thresholds varied by voxel size and tissue density and were adjusted by eye to create surfaces with reduced artifact, maximal bone quality, and high definition. Because these criteria are subjective, no single setting optimizes 3D surface reconstruction for all specimens. For consistency, the thresholds were determined by the same individual (S.S.) across all samples.

### 2.5 Landmarks

**FIGURE 1:**
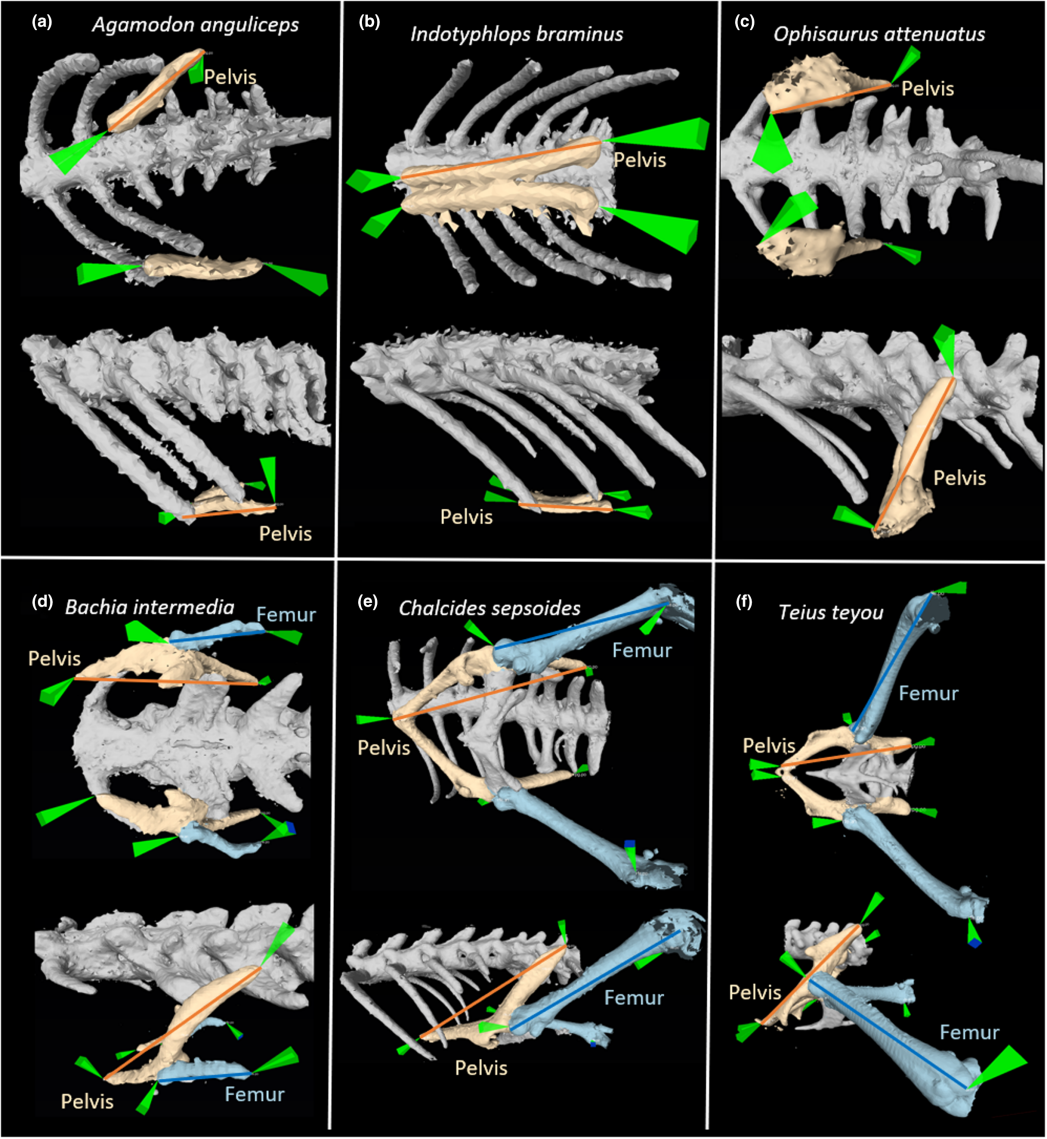
Ventral (top image in each panel) and lateral (bottom image in each panel) images showing the pelvis (tan) and femurs (blue) of representative specimens. (a) *Agamodon anguliceps* (pelvis only), (b) *Bachia intermedia* (pelvis plus femur), (c) *Indotyphlops braminus* (pelvis only), (d) *Ophisaurus attenuatuas* (pelvis only), (e) *Chalcides sepsoides* (pelvis plus femur), and (f) *Teius teyou* (pelvis plus femur)

Homologous landmarks were placed on each 3D surface using open‐source software MeshLab 2016. All landmarks were digitized by S.S. for consistency. Each image was rotated during landmark placement to minimize parallax, and the images were rotated after landmark placement to ensure visually that they had been placed correctly. We marked the anterior point of the pubis and the posterior point of the ilium on right and left sides to measure the pelvis (Figure 1). In specimens with a less well‐developed pubis, the anterior–medial point of the epipubis was used as the anterior landmark. In species without femurs, the pubis, ilium, and ischium bones of the pelvis were usually indistinguishable; we, therefore, landmarked the anterior and posterior points of the pelvic vestige.

For species with femurs, we placed landmarks on the proximal point of the femur head (at the hip) and the most distal point of the femur (at the knee; Figure 1). CT resolution was not fine enough to distinguish distal limb bones in *B. intermedia* due to extensive reduction (Figure 1b). Therefore, proximal landmarks were placed on femur head and distal landmarks were placed on the most distal skeletal point of the limb, which might include non‐femoral elements. However, in mice, altered *Pitx1* expression resulted in a reduction in the tibia, fibula, and metatarsals, as well as the pelvis and femur (Lanctôt et al., 1999; Marcil et al., 2003; Szeto et al., 1999). Thus, including elements distal to the femur should yield a suitable measure of hindlimb length in *B. intermedia*, especially since our metric is asymmetry. We exported landmarks from MeshLab as picked_points.pp files.

### 2.6 Analysis

Analysis was conducted in R and code is accessioned with our data on datadryad.org. We used a bespoke function called *read.pp* (Griffin, 2017) to extract 3‐D landmarks from picked_points.pp files. We then calculated the length in mm of each limb element (pelvis or femur) as the distance between landmarks using the *dist* function (method = “Euclidean”) in the *stats* package to two decimal places. We identified outliers with trait values more than 3 standard deviations from the mean, checked them against our MeshLab digitizations to see if they were caused by misplaced landmarks, and re‐digitized them, if necessary. Otherwise, outliers were removed from the analysis. Specimens with a broken femur or pelvis were excluded from the analysis.

Following Stuart et al. (2020), we calculated percent asymmetry for individuals by subtracting the left trait length from the right trait length, dividing by the sum of left and right lengths, and multiplying by 100, to two decimal places. A negative value for percent asymmetry, therefore, indicates that the left side was larger than the right side. We did not control for within‐species body size variation in our samples, assuming isometry between body size and percent asymmetry. We did not account for sex in our analyses, as these data were not consistently available for the museum specimens and could not be reliably scored without damaging them through dissection.

**FIGURE 2:**
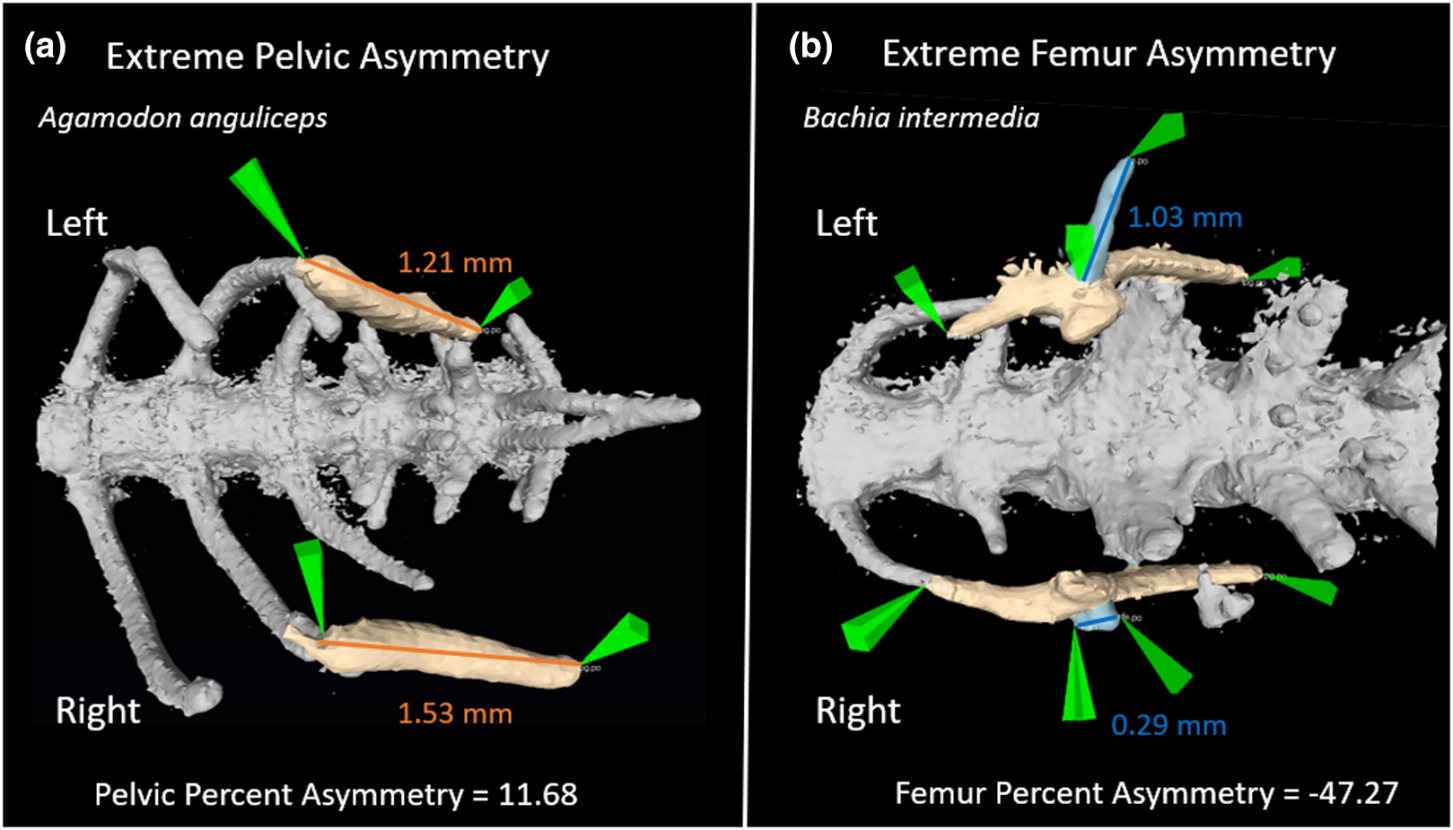
Ventral images showing the pelvis and femurs of the most asymmetrical specimens. (a) Most extreme percent asymmetry from the pelvic, *Agamodon anguliceps*, and (b) femur, *Bachia intermedia*

We used a two‐tailed chi‐square test (*chi.sq.test* in the *stats* package) to test for significant deviation from 50:50 frequencies of left‐ versus right‐larger trait asymmetry, separately for each taxon and trait, excluding symmetric individuals for the chi‐square test. This test only considers the sign of the left–right difference. To test for asymmetry in trait magnitude, we used a two‐tailed, paired t‐test (*t.test* in the *stats* package) to test whether left‐side vestige size was significantly different from right‐side vestige size (e.g., Figure 2).

## 3 RESULTS

**FIGURE 3:**
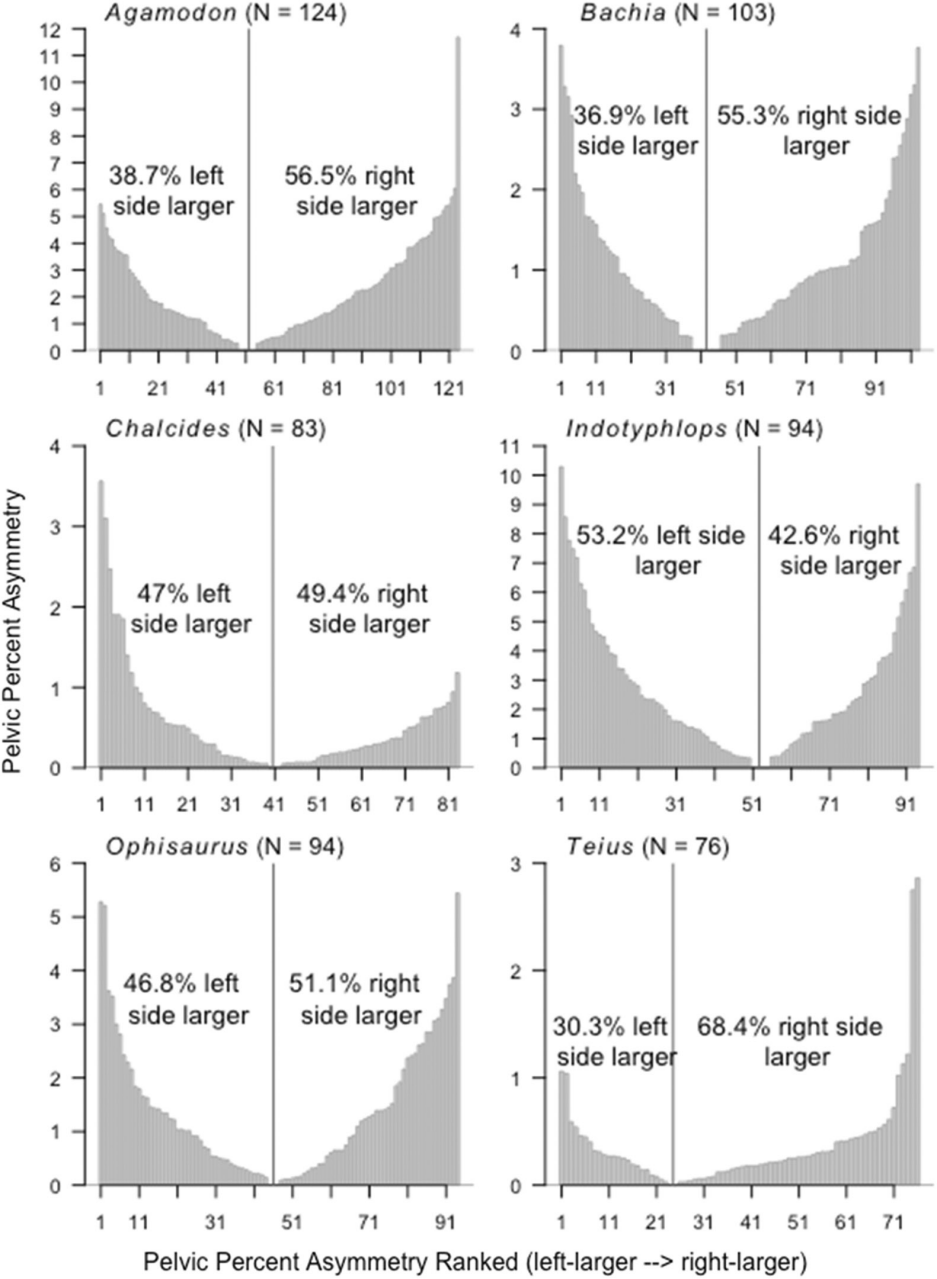
Plots showing the rank and absolute magnitude of larger‐left and larger‐right pelvic asymmetries. Each bar is an individual. Individuals on the left side of the vertical line have left‐larger pelvic asymmetry. The negative values indicating left‐larger asymmetry have been reflected across the *x*‐axis to facilitate comparison to right‐larger individuals plotted on the right side of the vertical line. Percentages of individuals with left‐ versus right‐larger vestiges do not add to 100% because of individuals with 0% asymmetry

**FIGURE 4:**
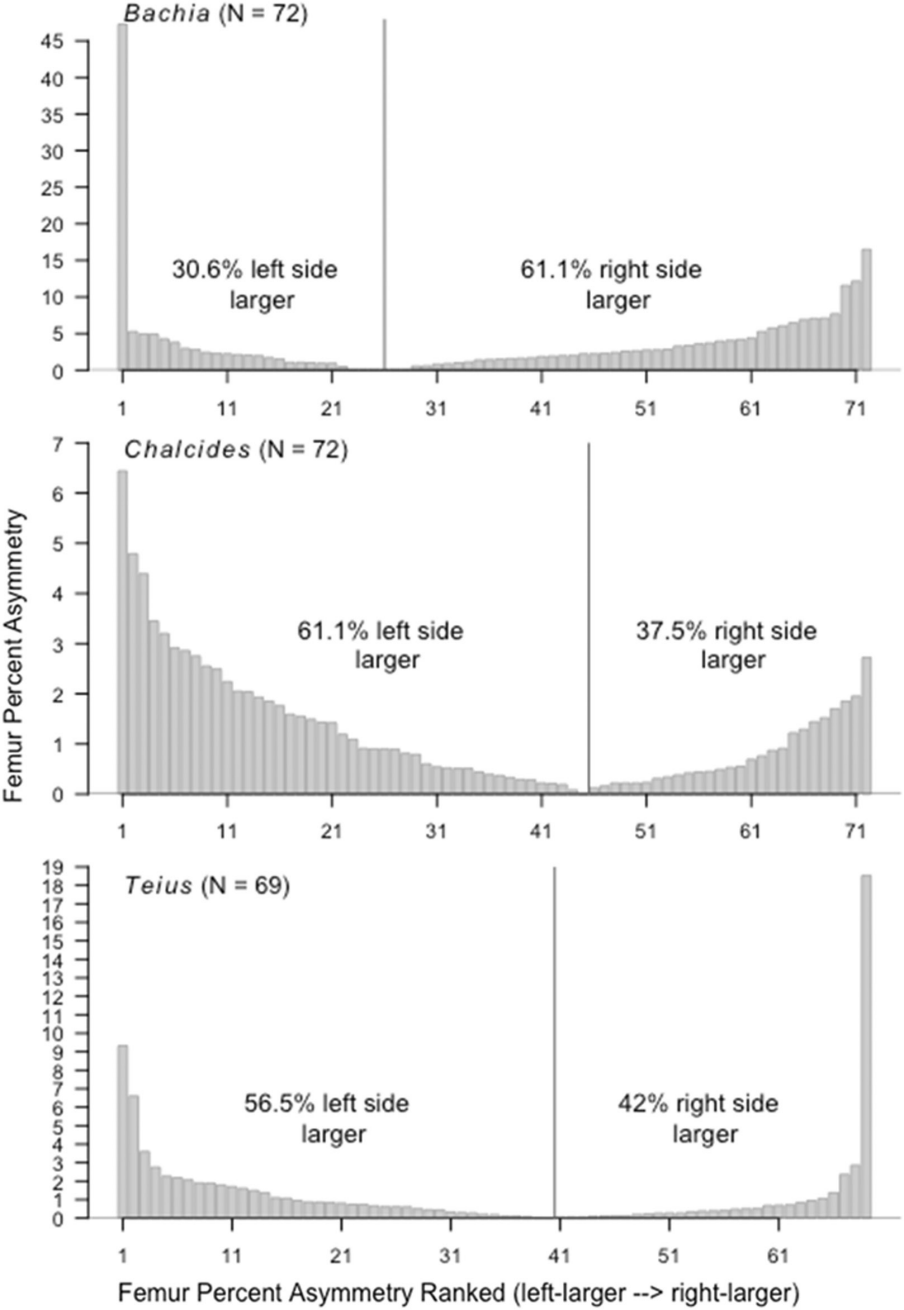
Plots showing the rank and absolute magnitude of larger‐left and larger‐right femur asymmetries. Each bar is an individual. Individuals on the left side of the vertical line have left‐larger femur asymmetry. The negative values indicating left‐larger asymmetry have been reflected across the *x*‐axis to facilitate comparison to right‐larger individuals plotted on the right side of the vertical line. Percentages of individuals with left‐ versus right‐larger vestiges do not add to 100% because of individuals with 0% asymmetry

Results are shown in Figures 3 and 4, with statistical output in Table 2. The pelvis and femur vestiges of *Chalcides sepsoides* were significantly larger on the left side, although the number of individuals with a larger left element did not deviate significantly from 50:50. *Bachia intermedia* had significantly more individuals with a larger right femur, and the right femur tended to be larger than the left femur; *Bachia* pelvises, however, did not deviate significantly from equal numbers of individuals with a larger‐left or larger‐right vestiges. *Teius teyou* had significantly more individuals with significantly larger right‐side pelvises, although no trends were detected in the femur. Otherwise, no significant asymmetry in frequency or magnitude was detected.

**TABLE 2:** Asymmetry in reduced pelvises and femurs

	*N*	*N*_left‐larger_	*N*_right‐larger_	$\chi_{1}^{2}$; *p*	% Asymmetry mean (SD)	Paired *t*_*N‐1*_ *; p*
(a) Pelvic asymmetry						
*Agamodon anguliceps*	124	48	70	1.71; .191	0.66 (2.72)	*2.54; .012*
*Bachia intermedia*	103	38	57	1.54; .215	0.20 (1.39)	1.41; .161
*Indotyphlops braminus*	94	50	39	0.46; .499	−0.43 (3.50)	−1.23; .223
*Ophisaurus* spp.	94	44	47	0.01; .941	0.12 (1.88)	0.57; .569
*Chalcides sepsoides*	83	39	41	0.00; 1.000	−0.18 (0.82)	**−2.11; .038**
*Teius teyou*	76	23	51	*4.74; .030*	0.18 (0.59)	*3.11; .003*
(b) Femur asymmetry						
*Bachia intermedia*	72	22	44	3.12; .077	0.96 (6.96)	1.86; .067
*Chalcides sepsoides*	72	44	27	1.61; .205	−0.64 (1.62)	**−3.37; .001**
*Teius teyou*	69	39	29	0.47; .492	−0.28 (2.85)	−1.03; .308

*Note*: Populations with negative percent asymmetry are left‐larger biased. Comparisons that are significantly left biased at the uncorrected *α* < 0.05 level are bold; right bias is indicated by italics. For some taxa, *N* is larger than the sum of *N*_left‐larger_ and *N*_right‐larger_ because some individuals had 0 asymmetry (per rounding error with two decimal places). Only the *Teius* pelvis and *Chalcides* femur *t*‐tests would survive multiple‐test correction (Bonferroni‐corrected α = 0.05/9 tests per statistical approach = 0.006).

For the above analyses, percent asymmetry was calculated from component bone lengths that were not deemed outliers. However, there were a few outliers among computed asymmetry values, suggesting an error in those cases. Post hoc, we reanalyzed the percent asymmetry values after removing percent asymmetry values greater than 3 standard deviations from mean asymmetry, by trait and taxon. Reanalysis without these outliers gave qualitatively similar results to those reported in Table 2 (results not shown).

## 4 DISCUSSION

Modified expression of *Pitx1* is responsible for pelvic reduction in many extant populations of Threespine and Ninespine Stickleback (Bell et al., 2007; Chan et al., 2010; Shapiro et al., 2004, 2006; Xie et al., 2019). The phenotypic signature of null *Pitx1* alleles—left‐larger directional asymmetry of pelvic vestiges—is observed in the Miocene stickleback *G. doryssus* (Stuart et al., 2020) and, notably, in manatees (Shapiro et al., 2006). These taxa are separated by tens to hundreds of millions of years of evolution, prompting our hypothesis that modifications of *Pitx1* expression could be a highly conserved route for the evolution of hind appendage reduction in vertebrates. We tested this hypothesis in squamates by testing for directional asymmetry in the frequencies and magnitudes of hindlimb vestiges in six squamate lineages that independently evolved hindlimb reduction (Greer, 1991; Wiens et al., 2006).

We found little evidence for repeated use of *Pitx1* in squamate hindlimb reduction. Left‐larger directional asymmetry was statistically supported only in the skink *Chalcides sepsoides* for the magnitude by which the pelvis and femur were larger on the left side—only 2 of the 18 contrasts that we made (Table 2). While this suggests the possible use of *Pitx1* in *Chalcides* (and might warrant further phylogenetic analysis of limb reduction across skinks), it is hardly resounding evidence for the repeated use of *Pitx1* in squamates. Indeed, *Pitx1* does not appear to be used within *Hemiergis*, a different genus of skinks in which expression of another transcription factor, *Sonic Hedgehog*, has been implicated in digit reduction (Shapiro et al., 2003; Swank et al., 2021).

Why might the squamate case be so different from that of the sticklebacks, in which *Pitx1* expression has repeatedly been modified independently in multiple populations across two species (Bell et al., 1985; Chan et al., 2010; Klepaker et al., 2013; Shapiro et al., 2004, 2006; Stuart et al., 2020; Xie et al., 2019)? First, perhaps not surprisingly, the probability that the same genes are used in parallel to obtain a repeated phenotype declines with divergence time (Conte et al., 2012). Stickleback lineages are young relative to the squamate lineages studied here and so might be more likely to show gene reuse.

Second, it appears that, although the coding sequence of *Pitx1* has a conserved role in teleost and tetrapod development, regulatory elements are less so. For example, in stickleback, knockouts to an upstream regulatory locus, *PelA*, have evolved repeatedly, likely because this enhancer is pelvis specific and is particularly vulnerable to deletion mutations (Chan et al., 2010; Xie et al., 2019). However, *PelA* is not found in tetrapods (Thompson et al., 2018).

*PelB* is another enhancer that can cause pelvic reduction in threespine stickleback. In transgenic stickleback, disruptions to *PelB* reduce *Pitx1* expression in the pelvic region and reduce the size of the pelvis, especially when *PelA* is no longer functional (Thompson et al., 2018). Unlike *PelA*, *PelB* is conserved in sequence and function between tetrapods and teleosts (Thompson et al., 2018), making it a possible locus for pelvic reduction in other vertebrates. However, loss of *PelB* function in transgenic mice results only in a partial reduction in *Pitx1* expression and a minor reduction in the long bones of the hindlimb autopod of transgenic mice (Thompson et al., 2018). These mutations have relatively small effects on both gene expression and bone length, resulting in only a 2–4% decrease in foot bone length (Thompson et al., 2018). Thus, *PelB* mutations alone would be unlikely to cause major reductions in squamates.

Tetrapods do have a hindlimb‐specific *Pitx1* enhancer, *Pen*, but disruptions of *Pen* could also affect forelimb development (Kragesteen et al., 2018). In wild‐type mice, *Pen* is partially responsible for *Pitx1* expression in the hindlimb, but a repressive, inactive chromatin structure prevents its expression in the forelimb (Kragesteen et al., 2018). Mutations in *Pen* that would reduce *Pitx1* expression in the hindlimb could also dysregulate *Pitx1* expression in the forelimb, leading to deleterious phenotypic effects (Kragesteen et al., 2018; Logan & Tabin, 1999; Minguillon et al., 2005; Thompson et al., 2018). Tetrapod evolution of hindlimb reduction based on *Pen* would therefore be constrained by negative pleiotropic effects on the anterior vertebrate appendage.

How, then, have manatees evolved left‐biased asymmetry in their pelvic vestiges (Shapiro et al., 2006)? Indeed, it was this observation that motivated the current study and Swank et al. (2021)—if manatees and stickleback have left‐biased pelvic asymmetry, might that pattern hold across more vertebrates? Apparently, it does not, or at least other genes are more important in squamates. Thus, molecular and developmental efforts to understand manatee pelvic asymmetry could reveal new genes that control symmetry in the body and new ways to evolve hindlimb reduction.

Significantly larger or more frequently larger vestiges on the right side were associated with squamate hindlimb reduction in two taxa (3 of 18 statistical comparisons at the 0.05 level; Table 2). These significant results may be due to chance in repeated comparisons. However, one comparison would survive multiple‐test correction (Table 2), and there is evidence from three geographically separated Alaskan populations of threespine stickleback of independent evolution of greater frequencies of individuals with a larger right pelvic vestige (Bell et al., 2007).

Right‐larger directional asymmetry is not unheard of in nature, and we propose a plausible model for the evolution of reduced pelvic skeletons with a larger right vestige. It is important that the right and left pelvic skeletons are bilaterally symmetrical to support normal function (i.e., locomotion and defense). When both *Pitx1* and *Pitx2* are expressed normally during development, a full, functional, and bilaterally symmetrical pelvic skeleton is produced because *Pitx1* and *Pitx2* should have counteracting asymmetric effects on pelvic skeleton development. When *Pitx1* is silenced and/or functionally disrupted experimentally, expression of *Pitx2* on the left side produces a larger left vestige (Chan et al., 2010; Gurnett et al., 2008; Kragesteen et al., 2018; Lanctôt et al., 1999; Marcil et al., 2003; Shapiro et al., 2004; Shapiro et al., 2006; Szeto et al., 1999; Thompson et al., 2018; Xie et al., 2019). Accordingly, we propose that if *Pitx2* has a large effect on the development of the pelvic skeleton, silencing it should allow the normal compensatory effect of *Pitx1* expression to produce larger right pelvic structures. Further research on *Pitx1* and *Pitx2* expression in threespine stickleback populations with right‐larger directional asymmetry or experimental studies that knock out the function of *Pitx1*, *Pitx2*, or both during pelvic development may provide insights into this possibility.

In conclusion, the limited evidence for use of altered *Pitx1* expression in hindlimb reduced squamates may result from the absence of the hindlimb‐specific enhancer, *PelA*, small effects of *PelB*, or the adverse pleiotropic effects of a disrupted *Pen* enhancer. Our findings are consistent with available evidence from non‐model organisms that there are multiple developmental and molecular routes to hindlimb reduction (Roscito et al., 2022; Swank et al., 2021). Consequently, hindlimb loss is likely non‐parallel in squamates and *Pitx1* is only one of the several genetic pathways that could be used to respond to similar selection pressures.

## AUTHOR CONTRIBUTIONS

**Samantha Swank:** Conceptualization (equal); data curation (lead); formal analysis (lead); validation (equal); visualization (equal); writing – original draft (lead); writing – review and editing (supporting). **Ethan Elazegui:** Data curation (equal); formal analysis (supporting); investigation (equal); validation (equal); visualization (equal); writing – original draft (supporting); writing – review and editing (supporting). **Sophia Janidlo:** Data curation (supporting); investigation (equal); methodology (equal). **Thomas J. Sanger:** Conceptualization (supporting); writing – review and editing (supporting). **Michael A Bell:** Conceptualization (equal); writing – review and editing (supporting). **Yoel E. Stuart:** Conceptualization (lead); formal analysis (equal); funding acquisition (lead); resources (lead); supervision (equal); validation (equal); visualization (equal); writing – original draft (equal); writing – review and editing (equal).

## CONFLICT OF INTEREST

The authors involved in the preparation of this manuscript have no conflicts of interest to declare.

### OPEN RESEARCH BADGES

This article has earned an Open Data badge for making publicly available the digitally‐shareable data necessary to reproduce the reported results. The data is available at doi: https://doi.org/10.5061/dryad.gb5mkkwsb.

## Supporting information


Supplementary Information

## Data Availability

The data and code needed to reproduce the analyses reported here will be archived on https://doi.org/10.5061/dryad.gb5mkkwsb. The raw, 3D‐μCT image data are archived at morphosource.org, under the collection name Squamate Reptile Hindlimb Asymmetry (https://www.morphosource.org/projects/000408092/about?locale=en).
